# Position and orientation measurement adopting camera calibrated by projection geometry of Plücker matrices of three-dimensional lines

**DOI:** 10.1038/srep44092

**Published:** 2017-03-07

**Authors:** Guan Xu, Anqi Zheng, Xiaotao Li, Jian Su

**Affiliations:** 1 Traffic and Transportation College, Nanling Campus, Jilin University, Renmin Str. 5988#, Changchun, China; 2 School of Mechanical Science and Engineering, Nanling Campus, Jilin University, Renmin Str. 5988#, Changchun, China

## Abstract

A position and orientation measurement method is investigated by adopting a camera calibrated by the projection geometry of the skew-symmetric Plücker matrices of 3D lines. The relationship between the Plücker matrices of the dual 3D lines and the 2D projective lines is provided in two vertical world coordinate planes. The transform matrix is generated from the projections of the 3D lines. The differences between the coordinates of the reprojective lines and the coordinates of extracted lines are employed to verify the calibration validity. Moreover, the differences between the standard movement distance of the target and the measurement distance are also presented to compare the calibration accuracy of the 3D line to 2D line method and the point-based method. Furthermore, we also explore the noise immunity of the two methods by adding Gaussian noises. Finally, an example to measure the position and orientation of a cart is performed as an application case of this method. The results are tabled for the reproduction by the readers. The results demonstrate that the line to line method contributes higher calibration accuracy and better noise immunity. The position and orientation measurement adopting the line to line method is valid for the future applications.

Camera is an important measurement instrument as it bridges the scales from the 3D space to the 2D space^1^,^2^. Camera calibration is the bridge to estimate the transformation matrix of the camera from a captured photograph^3^. Consequently, the camera calibration is widely studied in the vision measurement and optical inspection, such as object reconstruction^4^, computed tomography^5^, pose estimation^6^, and robot arm positioning^7^. As the transformation matrix of the camera contains the position and orientation information of a measured object in a captured image, we focus on the position and orientation measurement technique using a calibrated camera by the projection geometry of Plücker matrices of three-dimensional lines.

Various methodologies have been explored to solve the camera calibration problem. These technologies are approximately classified by the methods based on 3D, 2D, and 1D calibration targets. The 1D target is firstly described by Zhang^8^. The target should rotate to a fixed point in the calibration. Qi^9^ introduced a calibration method using the 1D object with three or more markers. The constraint equations of the camera parameters are provided by the rotation around one marker which is moving in a plane. 1D calibration method provides simple structure and easy operation. However, the accuracy of 1D calibration methods is generally low due to the insufficient information on the 1D bar. Consequently, many calibration methods are mainly based on the 3D or 2D targets. To promote the camera calibration accuracy, Ricolfeviala^10^ proposes an optimal calibration method based on several images of a 2D pattern. The optimal conditions are proposed to resolve the calibration process accurately. Bethea^11^ develops a camera calibration technique by employing three parallel calibration planes and two cameras. Heikkila^12^ presents an approach to calibrate the camera by circular control points identified on two perpendicular planes. In this paper, 3D target is chosen to calibrate the camera due to the high calibration accuracy and the sufficient information of the calibration target.

Various patterns are employed on the calibration objects, such as points, circles, lines, and color patterns. Most of the calibrations adopt the feature points of the target^13^,^14^. Point-based calibration method achieves the advantages of high speed and easy operation. However, it is easily affected by the image noises. The circle-pattern-based calibration technique also attracts many investigators due to the high noise immunity. Xue^15^ describes a method using concentric circles and wedge grating for camera calibration. An improved calibration method is proposed by Rui^16^ to increase the camera and projector calibration accuracy simultaneously by detecting the edge of the circles. Xu^17^ investigates a camera calibration method using the perpendicularity of 2D lines in the target observations. A study is presented by Yilmaztürk^18^ for full automatic calibration of color digital cameras using color targets. Nevertheless, the color distortion is an unavoidable element in the process of capturing the color photos. Although the circle-pattern-based calibrations contribute high noise immunity, the method shows low efficiency due to the low speed of extracting the circles. The line-pattern-based calibration method is selected in this paper considering the moderate speed of extracting lines and good noises immunity. The original line-pattern-based calibration method employs the geometry relationship between the 2D line on a planar calibration target and the 2D projective line in the image. The essence of the method above is a 2D line to 2D line homography. The 3D calibration target is chosen to calibrate the camera owing to the high accuracy. However, it is difficult to build the homography from the 3D line on the target to the 2D line in the image as the coordinates of a 3D line are generally indicated by the equations of two planes. Therefore, there is a lack of the calibration method adopting the projective geometry from 3D line to 2D projective line.

In the paper, the position and orientation of an object are obtained from the captured image of a calibrated camera. Therefore, we firstly explore the camera calibration method adopting the projection geometry from the Plücker matrices of 3D lines to the 2D projective lines. A projective line in the image is determined by the corresponding 3D line on the calibration target and the projective plane. The transformation matrix of the camera is generated from the geometrical relationship between 3D lines and 2D projective lines. The 3D line to 2D line method is compared with the point-based method to verify the measurement validity, the measurement accuracy and the noise immunity. Then, a cart with the 3D target is chosen as the application example. The transformation matrix of the camera is decomposed to the rotation matrix, translation vector and the intrinsic matrix. The position and orientation of the measured cart is generated from the translation vector and the rotation matrix, and verified by the absolute and relative errors of the reconstructed displacements.

## Results

**Figure 1:**
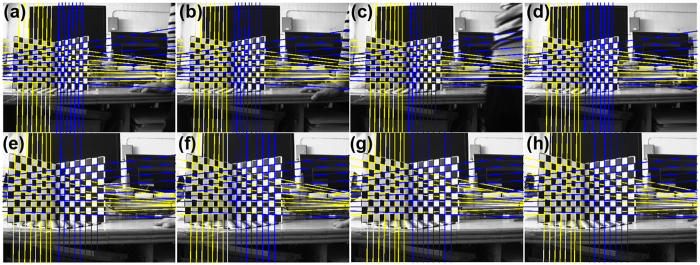
Experiment results of line extraction. (**a**–**d**) Are the line recognition results in the first group of experiments as the movement distances are 10 mm 20 mm 30 mm and 40 mm, respectively. (**e**–**h**) Are the line recognition results in the second group of experiments as the movement distances are 10 mm 20 mm 30 mm and 40 mm, respectively.

According to the 3D line to 2D line method, the transformation matrix P is generated from *n* 3D lines L_*i*_ and *n* 2D projective lines **l**_***i***_. The coordinates of the 2D projective lines are extracted by the Hough transform^19^. The recognition results of the lines are shown in Fig. 1. The results indicate the Hough transform can extract the lines exactly.

**Figure 2:**
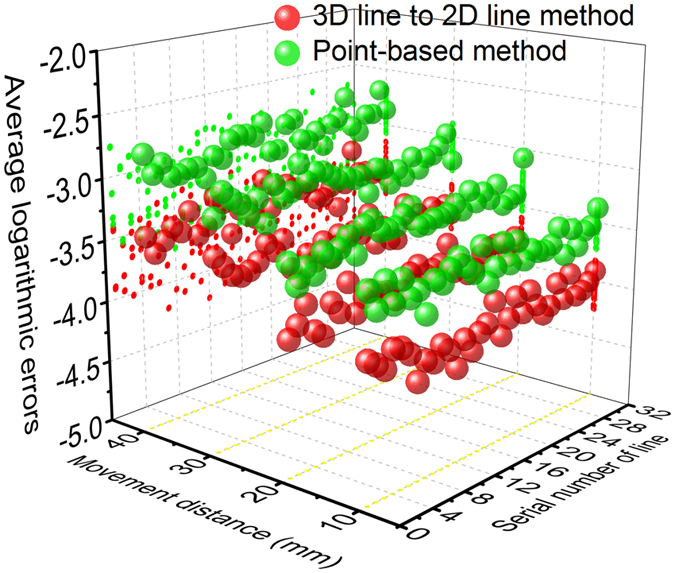
The average logarithmic errors related to the movement distance and the line, in the 3D line to 2D line method and point-based method in the first group of experiments.

The differences between the coordinates of the reprojective lines and the line coordinates extracting by the Hough transform are employed to evaluate the accuracy of the 3D line to 2D line method. The comparison of the average logarithmic errors adopting the 3D line to 2D line method and the point-based method^20^ is shown in Fig. 2. The image resolution is 1024 × 768. Moreover, in order to explore the relationship between the errors and the movement distance, the calibration board is moved by 10 mm, 20 mm, 30 mm, and 40 mm respectively. In the first group of experiments, the images are captured at the measurement distance of 1000 mm. The mean of average logarithmic errors using the line to line method are 1.11 × 10^−4^, 1.67 × 10^−4^, 2.78 × 10^−4^, and 4.09 × 10^−4^ corresponding to the movement distances of 10 mm, 20 mm, 30 mm, and 40 mm. The mean of average logarithmic errors of the point-based method is 3.19 × 10^−4^, 5.11 × 10^−4^, 8.55 × 10^−4^, and 1.41 × 10^−3^ corresponding to the movement distances of 10 mm, 20 mm, 30 mm, and 40 mm.

**Figure 3:**
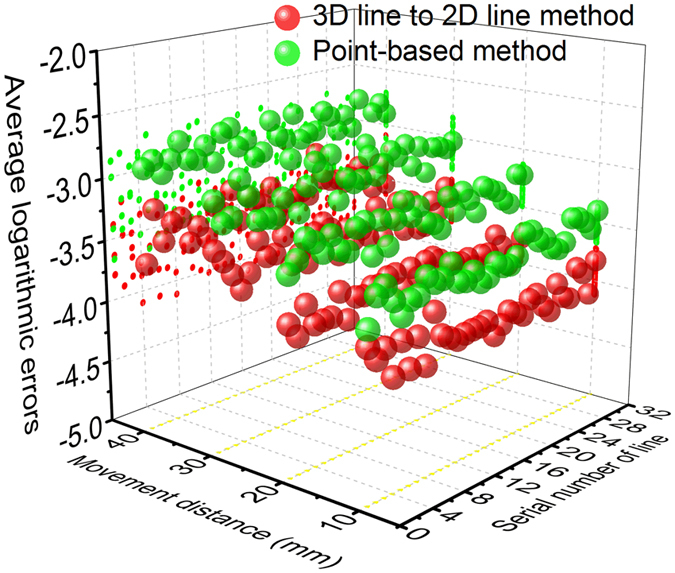
The average logarithmic errors related to the movement distance and the line, in the 3D line to 2D line method and point-based method in the second group of experiments.

Besides, the second group of experiments are performed at a smaller measurement distance. The images are observed by the camera at the measurement distances of 800 mm. Similar to first group of experiments, the 3D line to 2D line method is compared with the point-based method to verify the measurement accuracy in Fig. 3. The mean of average logarithmic errors using the line to line method are 1.01 × 10^−4^, 1.61 × 10^−4^, 2.50 × 10^−4^, and 3.99 × 10^−4^ corresponding to the movement distances of 10 mm, 20 mm, 30 mm, and 40 mm. The mean of average logarithmic errors adopting point-based method are 3.12 × 10^−4^, 4.81 × 10^−4^, 8.08 × 10^−4^, and 1.34 × 10^−3^ corresponding to the movement distance of 10 mm, 20 mm, 30 mm, and 40 mm. The results of the two groups of experiments both show that the errors increase with the increasing movement distance. Moreover, the errors of the 3D line to 2D line method are all smaller than the point-based method at the movement distances. The results indicate that the line to line method provides higher calibration accuracy. Furthermore, the errors of the images at the distance of 800 mm are smaller than the errors of the images at the distance of 1000 mm. The two methods achieve higher measurement accuracy in the small measurement distance.

**Figure 4:**
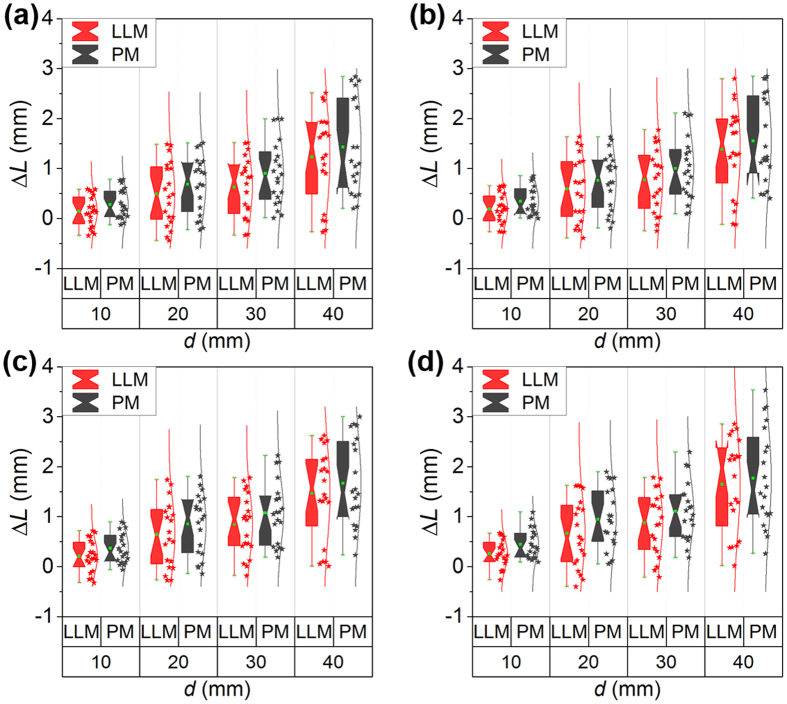
The measurement errors of the 3D line to 2D line method (LLM) and the point-based method (PM) in the first group of experiments. (**a**) Is the measurement errors without noises. (**b**–**d**) Are the measurement errors with noises of 0.001, 0.005, and 0.01, respectively.

**Figure 5:**
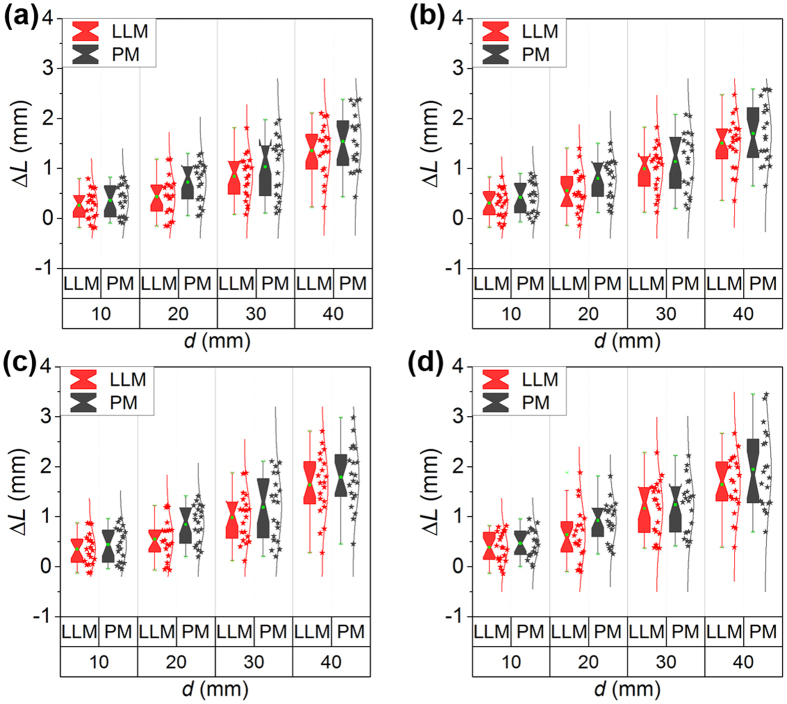
The measurement errors of the 3D line to 2D line method (LLM) and the point-based method (PM) in the second group of experiments. (**a**) Is the measurement errors without noises. (**b**–**d**) Are the measurement errors with noises of 0.001, 0.005, and 0.01, respectively.

Furthermore, three levels of Gaussian noises are added to study the effects of the noises. The 3D line to 2D line calibration method is also compared with the point-based method in the two groups of experiments. The measurement errors are evaluated by


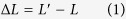


where *L′* is the reconstructed movement distance of the calibration board from the first place to the next place. *L* is the standard movement distance. *L′* is generated from P. The standard distances are 10 mm, 20 mm, 30 mm, and 40 mm, respectively. We perform 20 experiments at the standard distances. The results of two groups of experiments are shown in Figs 4 and 5, respectively. In the first group of experiments, the means of Δ*L* adopting the 3D line to 2D line method without noises are 0.26 mm, 0.43 mm, 0.84 mm, and 1.35 mm when the movement distances are 10 mm, 20 mm, 30 mm, and 40 mm. The corresponding errors of the point-based method are 0.36 mm, 0.72 mm, 1.03 mm, and 1.54 mm, respectively. The mean errors using the point-based method are evidently bigger than the 3D line to 2D line method. When the noise level is 0.0001, the mean errors adopting the line to line method are 0.31 mm, 0.56 mm, 0.99 mm, and 1.51 mm with respect to the movement distances of 10 mm, 20 mm, 30 mm, and 40 mm. The mean errors of the point-based method are 0.42 mm, 0.80 mm, 1.14 mm, and 1.70 mm. The mean errors of the line to line method under the noise of 0.005 are 0.35 mm, 0.55 mm, 0.98 mm, and 1.63 mm when the movement distances are 10 mm, 20 mm, 30 mm, and 40 mm, respectively. The mean errors of the point-based method under the noise level of 0.0005 are 0.44 mm, 0.84 mm, 1.18 mm, and 1.79 mm, respectively. The mean errors of the line to line method under the noise of 0.01 are 0.39 mm, 0.64 mm, 1.16 mm and 1.64 mm as the movement distance increases from 10 mm to 40 mm. The mean errors using the point-based method are 0.46 mm, 0.92 mm, 1.24 mm, and 1.95 mm.

The second group of experiments are carried out in the measurement distance of 800 mm. The mean Δ*L* of the 3D line to 2D line method without noises are 0.14 mm, 0.49 mm, 0.63 mm, and 1.23 mm with the movement distances of 10 mm, 20 mm, 30 mm, and 40 mm. The related mean errors of the point-based method are 0.28 mm, 0.68 mm, 0.90 mm, and 1.43 mm, respectively. The mean errors of the line to line method are smaller than the point-based method. When the 0.0001 noise is added, the mean errors of the 3D line to 2D line method are 0.19 mm, 0.59 mm, 0.77 mm, and 1.38 mm corresponding to the movement distances of 10 mm, 20 mm, 30 mm, and 40 mm. The mean errors of the point-based method are 0.34 mm, 0.76 mm, 1.00 mm, and 1.55 mm. The mean errors of the line to line method under the noise level of 0.005 are 0.21 mm, 0.65 mm, 0.84 mm, and 1.46 mm when the movement distance are 10 mm, 20 mm, 30 mm, and 40 mm, respectively. The mean errors of the point-based method under the noise of 0.0005 are 0.37 mm, 0.85 mm, 1.07 mm, and 1.67 mm when the movement distances are 10 mm, 20 mm, 30 mm, and 40 mm, respectively. The mean errors of the 3D line to 2D line method under the noise level of 0.01 are 0.25 mm, 0.67 mm, 0.88 mm and 1.65 mm as the movement distance grows from 10 mm to 40 mm. The mean errors using the point-based method are 0.44 mm, 0.95 mm, 1.11 mm, and 1.77 mm, respectively.

**Figure 6:**
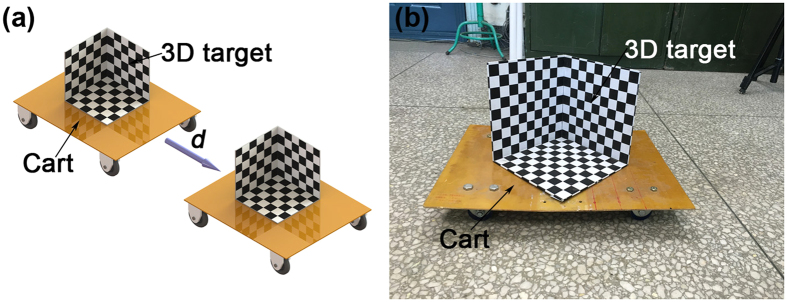
The measurement example of the position and orientation of a cart attached with a 3D target. (**a**) The cart is moved with the displacements *d* of 10 mm, 20 mm, 30 mm, 40 mm, respectively. (**b**) The experiment image of the position and orientation measurement.

In order to explain the application on how the technique can be used, a clear example is provided to measure the position and orientation of a cart. The details of the example are illustrated in Fig. 6, in which a cart is attached by a 3D target on the top and translated with the displacements of 10 mm, 20 mm, 30 mm and 40 mm, respectively.

**Figure 7:**
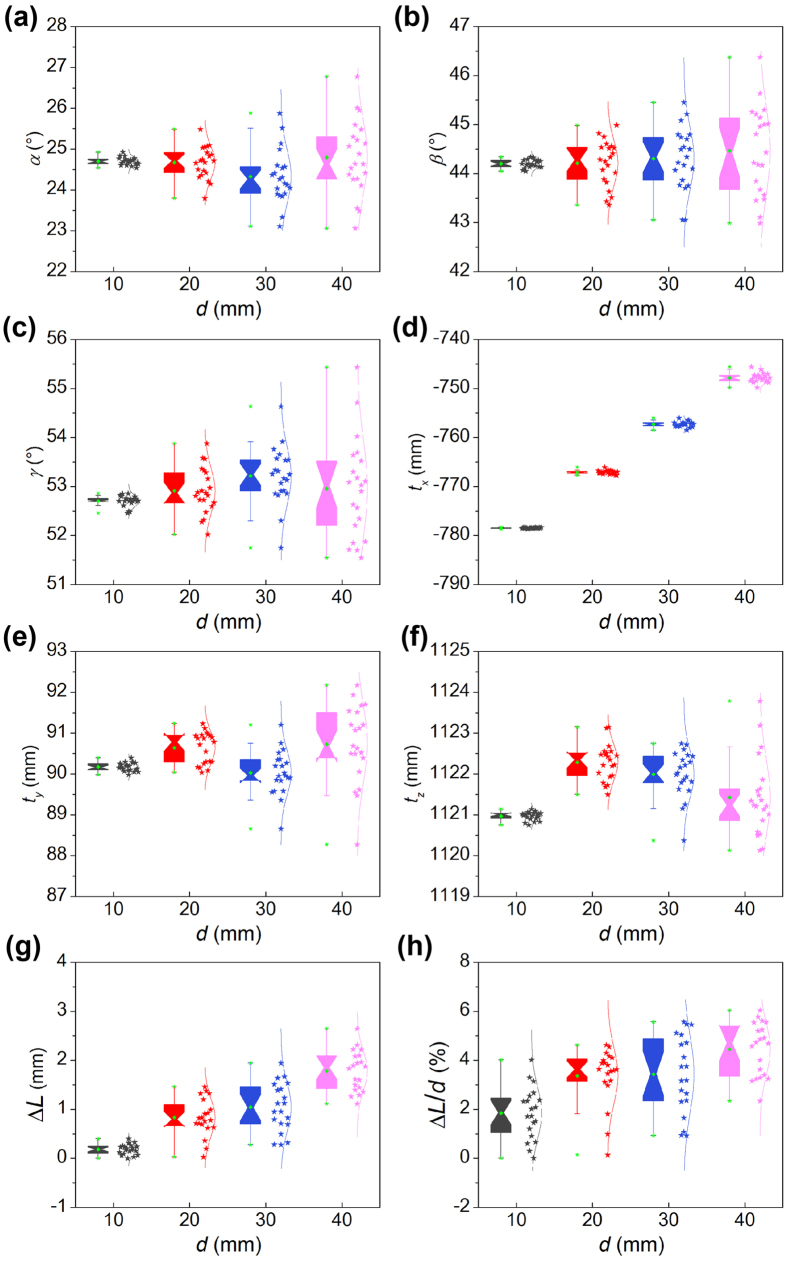
The measurement results of the position and orientation of a cart attached with a 3D target. (**a–c**) Are the rotation angles *α, β, γ* about the *o-x, o-y, o-z* axes of the camera coordinate system. (**d–f)** Are the translations *t*_*x*_, *t*_*y*_, *t*_*z*_ along the *o-x, o-y, o-z* axes of the camera coordinate system. (**g**) The absolute errors of the reconstructed displacements. (**h**) The relative errors of the reconstructed displacements.

The positions *t*_*x*_, *t*_*y*_, *t*_*z*_ and orientations *α, β, γ* of the cart about the *o-x, o-y, o-z* axes of the camera coordinate system in 20 different places are shown in Fig. 7(a–f). As the orientations *α, β, γ* are stable in the three angles in Fig. 7(a–c), the cart is moved along a straight line. According to the position data of the cart in Fig. 7(d–f), the displacement of the cart can be solved by the norm of the difference between the translation vector at the first place and the translation vector at the second place in the camera coordinate system. The absolute and relative errors of the reconstructed displacements in Fig. 7(g–h) are considered as the indicators to verify the measurement results of the cart. The measurement results are listed in Table 1.

As the movement displacement is 10 mm, the maximums of translations *t*_*x*_, *t*_*y*_, *t*_*z*_ are −778.28 mm, 90.41 mm and 1121.14 mm, respectively. The minimums of translations *t*_*x*_, *t*_*y*_, *t*_*z*_ are −778.64 mm, 89.98 mm and 1120.76 mm, respectively. The maximums of rotation angles *α, β, γ* are 24.92°, 44.34° and 52.86°, respectively. The minimums of rotation angles *α, β, γ* are 24.54°, 44.05°, and 52.45°, respectively. The means of measurement errors Δ*L* and relative errors Δ*L*/*d* are 0.18 mm and 1.85%, respectively. When the movement displacement increases to 40 mm, the maximal translations *t*_*x*_, *t*_*y*_, *t*_*z*_ are −745.61 mm, 91.17 mm and 1123.78 mm, respectively. The minimal translations *t*_*x*_, *t*_*y*_, *t*_*z*_ are −749.82 mm, 88.27 mm and 1120.13 mm, respectively. The maximal rotation angles *α, β, γ* are 26.77°, 46.37° and 55.43°, respectively. The minimal rotation angles *α, β, γ* are 23.06°, 42.99°, and 51.55°, respectively. The means of measurement errors and relative errors grow to 1.78 mm and 4.45%, respectively.

According to the above analysis and data in Table 1, since the translations *t*_*y*_, *t*_*z*_ do not vary obviously, the *o-x* direction of the translation *t*_*x*_ is the major movement direction. Moreover, the rotation angles about the three axes vary a little due to the cart is moved by a straight line. The mean of measurement errors increases with the rising movement displacement. The relative error also increases as the displacement is on the rise. Finally, the relative errors are less than 5% in most cases of experiments. It reveals that the measurement method is valid in the applications to solve the orientation and the position of an object.

## Discussion

According to the analysis above, the mean errors of the 3D line to 2D line method and the point-based method grow with the increasing noises. The mean errors of the 3D line to 2D line method are smaller than the point-based method. In the test without noises, the line to line method achieves the maximum relative error of 7.42% and minimum relative error of 0.68%. The ones of the point-based method are 7.83% and 1.13%, respectively. In the test with the noises, the line to line method provides the maximum relative error of 8.14% and minimum relative error of 0.30%. The ones of the point-based method are 8.17% and 0.55%, respectively. The results show that the 3D line to 2D line method contributes higher noise immunity. Moreover, the errors in the 800 mm measurement distance are smaller than the ones in the 1000 mm measurement distance in the experiments. The data reveal that the two calibration methods provide higher noise immunity in the near-camera measurement. In the example to measure the position and orientation the of the object, the proposed method achieves the error means of 0.18 mm, 0.83 mm, 1.05 mm and 1.78 mm corresponding to the measurement displacements of 10 mm, 20 mm, 30 mm and 40 mm. It indicates that the method is workable and reliable in the measurement applications of the position and orientation.

## Methods

**Figure 8:**
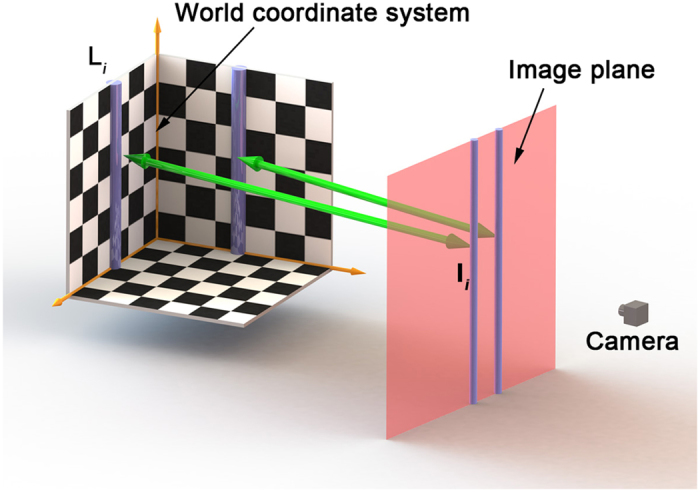
The calibration method adopts 3D lines L_*i*_ and 2D projective lines l_*i*_.

The calibration method is interpreted in the Fig. 8. A 3D line L_*i*_ on the 3D target is projected to the 2D image plane. The projective line **l**_*i*_ in the image plane is denoted by


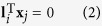


where **l**_*i*_ = (*l*_1*i*_, *l*_2*i*_, 1)^T^ is the homogenous coordinate of the projected line in the image plane, **x**_*j*_ = (*x*_1*j*_, *x*_2*j*_, 1)^T^ is the homogenous coordinate of the points on the projective line **l**_*i*_.

The projective plane **Q**_*i*_, which passes through the camera center and the 2D projective line **l**_*i*_, is determined by ref. 21


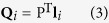


where P = [*p*_*mn*_]_3×4_ is the projective matrix of the camera. **l**_*i*_ = [*l*_*i*1_, *l*_*i*2_, 1]^T^ is the vector of the projective line.

The 3D crossing line between the projective plane **Q**_*i*_ and the *O*-*XZ* plane of the target can be expressed by the Plücker matrix^21^ as


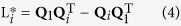


where **Q**_1_ = [0, 1, 0, 0]^T^ is the vector of the *O*-*XZ* plane of the target.

Substituting equation (3) in equation (4), the 3D crossing line is


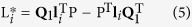


Equation (5) discloses the relationship between a 3D line 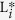 and its 2D projective line **l**_*i*_. According to the definitions of **Q**_1_, **l**_*i*_ and P, we have


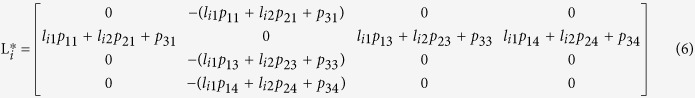


In the other way, the dual 3D line of the 3D line 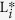 is defined by the Plücker matrix^21^ as


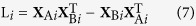


where **X**_A*i*_ = [*x*_A*i*_, 0, *z*_A*i*_, 1]^T^, **X**_B*i*_ = [*x*_B*i*_, 0, *z*_B*i*_, 1]^T^ are the vectors of 3D points A_*i*_, B_*i*_ on the 3D line L_*i*_ of the target. Then, we have


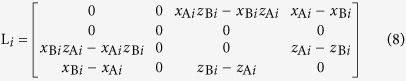


Considering the relationship between the 3D line 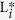 and its dual **L**_*i*_ as ref. 22


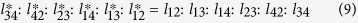


The 3D line 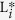 can also be derived from


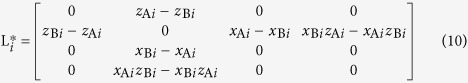


From equations (6) and (10), we have


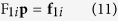


where 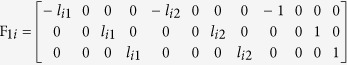, **p** = [*p*_11_, *p*_12_, *p*_13_, *p*_14_, *p*_21_, *p*_22_, *p*_23_, *p*_24_, *p*_31_, *p*_32_, *p*_33_, *p*_34_]^T^, **f**_1*i*_ = [*z*_A*i*_ − *z*_B*i*_, *x*_A*i*_ − *x*_B*i*_, *x*_B*i*_*z*_A*i*_ − *x*_A*i*_*z*_B*i*_]^T^.

In a similar way, the Plücker matrices of the 3D lines on the *O*-*YZ* plane of the target provide


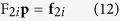


where 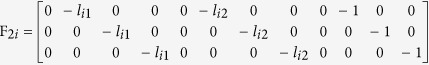, **f**_2*i*_ = [*z*_C*i*_ − *z*_D*i*_, *y*_D*i*_ − *y*_C*i*_, *y*_C*i*_*z*_D*i*_ − *z*_C*i*_*y*_D*i*_]^T^.

The stacking of equations (11) and (12) is


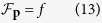


where 
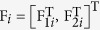
, 
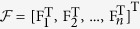
, 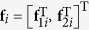, 
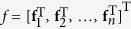
.

The non-homogeneous linear equations are solved by


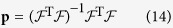


According to the vector **p** solved by equation (14), projection matrix P = [*p*_*mn*_]_3×4_ is the obtained and can be denoted by its decomposition as ref. 21


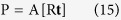


where A is the intrinsic matrix of the camera, R = [*r*_*mn*_]_3×3_ is the rotation matrix and **t** = [*t*_*x*_, *t*_*y*_, *t*_*z*_]^T^ is the translation vector from the world coordinate system defined in the 3D target and the camera coordinate system.

The translation vector **t** and the rotation matrix R provide the position and orientation of the measured object. Considering the orthogonality of the rotation matrix R and the upper triangular matrix A, the position and orientation are solved by ref. 23


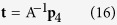



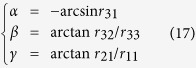


where **p**_4_ is the fourth column of the projection matrix P, *α, β, γ* are the rotation angles about the *o-x, o-y, o-z* axes of the camera coordinate system, *r*_11_, *r*_21_, *r*_31_, *r*_32_, *r*_33_ are the corresponding elements in the rotation matrix R = [*r*_*mn*_]_3×3_.

## Additional Information

**How to cite this article**: Xu, G. *et al*. Position and orientation measurement adopting camera calibrated by projection geometry of Plücker matrices of three-dimensional lines. *Sci. Rep.* **7**, 44092; doi: 10.1038/srep44092 (2017).

**Publisher's note:** Springer Nature remains neutral with regard to jurisdictional claims in published maps and institutional affiliations.

## Figures and Tables

**Table 1:** The means, the variances, the maximums and the minimums of the rotation angles *α, β, γ*, the translations *t*_*x*_, *t*_*y*_, *t*_*z*_, the absolute errors and relative errors of the reconstructed displacements.

*d* (mm)		*t*_*x*_ (mm)	*t*_*y*_(mm)	*t*_*z*_(mm)	*α* (°)	*β* (°)	*γ* (°)	Δ*L* (mm)	Δ*L/d* (%)
10	Mean	−778.46	90.17	1120.97	24.71	44.22	52.7	0.18	1.85
	Variance	0.085	0.11	0.13	0.09	0.081	0.11	0.11	1.05
	Maximum	−778.28	90.41	1121.14	24.92	44.34	52.86	0.41	4.08
	Minimum	−778.64	89.98	1120.76	24.54	44.05	52.45	0.01	0.1
20	Mean	−767.1	90.64	1122.28	24.67	44.21	52.92	0.83	3.37
	Variance	0.36	0.38	0.43	0.39	0.47	0.47	0.38	1.89
	Maximum	−766.07	91.23	1123.15	25.48	44.98	53.88	1.46	7.28
	Minimum	−767.71	90.04	1121.45	23.79	43.36	52.02	0.03	0.16
30	Mean	−757.32	90.03	1121.99	24.33	44.31	53.22	1.05	3.43
	Variance	0.56	0.54	0.58	0.65	0.64	0.59	0.5	1.57
	Maximum	−756.01	91.2	1122.74	25.88	45.45	54.63	1.94	6.47
	Minimum	−758.48	88.66	1120.37	23.11	43.06	51.75	0.28	0.93
40	Mean	−747.81	90.73	1121.43	24.79	44.47	52.96	1.78	4.45
	Variance	0.94	0.96	0.95	0.89	0.91	1.01	0.41	1.08
	Maximum	−745.6	92.17	1123.78	26.77	46.37	55.43	2.65	6.63
	Minimum	−749.82	88.27	1120.13	23.06	42.99	51.55	1.11	2.78
